# Recruitment, outcomes, and toxicity trends in phase I oncology trials: Six‐year experience in a large institution

**DOI:** 10.1002/cnr2.1465

**Published:** 2021-07-10

**Authors:** Siddharth Menon, Amy Davies, Sophia Frentzas, Cheryl‐Ann Hawkins, Eva Segelov, Daphne Day, Ben Markman

**Affiliations:** ^1^ Monash Health Melbourne Australia; ^2^ Olivia Newton‐John Cancer Research Institute Melbourne Australia; ^3^ La Trobe University Melbourne Australia; ^4^ Monash University Melbourne Australia; ^5^ The Alfred Hospital Melbourne Australia

**Keywords:** immunotherapy, phase one trials, response

## Abstract

**Background:**

With the rapid influx of novel anti‐cancer agents, phase I clinical trials in oncology are evolving. Historically, response rates on early phase trials have been modest with the clinical benefit and ethics of enrolment debated. However, there is a paucity of real‐world data in this setting.

**Aim:**

To better understand the changing landscape of phase I oncology trials, we performed a retrospective review at our institution to examine patient and trial characteristics, screening outcomes, and treatment outcomes.

**Methods and results:**

We analyzed all consecutive adult patients with advanced solid organ malignancies who were screened across phase I trials from January 2013 to December 2018 at a single institution. During this period, 242 patients were assessed for 28 different trials. Median age was 64 years (range 30–89) with an equal sex distribution. Among 257 screening visits, the overall screen failure rate was 18%, resulting in 212 patients being enrolled onto a study. Twenty‐six trials (93%) involved immunotherapeutic agents or molecular targeted agents either alone or in combination, with only two trials of cytotoxic agents (7%). Twenty‐two (13.4%) of the 209 treated patients experienced a total of 33 grade 3 or higher treatment‐related adverse events. There was one treatment‐related death (0.5%). Of 190 response‐evaluable patients, 7 (4%) had a complete response, 34 (18%) a partial response, and 59 (31%) experienced stable disease for a disease control rate of 53%. The median overall survival for our cohort was 8.0 (95% CI: 6.8–9.2) months.

**Conclusion:**

The profile of phase I trials at our institution are consistent with the changing early drug development landscape. Response rates and overall survival in our cohort are superior to historically reported rates and comparable to contemporaneous studies. Severe treatment‐related toxicity was relatively uncommon, and treatment‐related mortality was rare.

## 1 INTRODUCTION

Phase I trials represent a crucial step wherein a novel therapeutic agent makes the transition from the pre‐clinical to clinical stage, thus providing a foundation for a potentially successful drug development program.^1^ These studies involve the early exploration of treatments or treatment combinations in humans. Determination of safety and tolerability is the primary objective, as well as establishing the maximum tolerated dose and/or the recommended phase II dose.^2^ However, early phase trials in oncology historically have had low success rates, with the chance of eventual approval for a tested drug being 7%—the lowest among all medical specialties as reported in a 2014 survey.^1^, ^3^ Additionally, previously reported clinical outcomes including low response rates (4%–10%), poor overall survival (OS, 5–6 months), and modest disease control rates (DCR, 20%–25%) have brought into question the therapeutic appeal and ethical justification of phase I trial enrolment.^4^, ^5^, ^6^

Nevertheless, the landscape of early phase oncology trials is changing. A meta‐analysis of phase I trials conducted between 2014 and 2015 demonstrated encouraging response rates of 20%.^7^ Trials that used an enrichment design (specific tumor type or biomarker driven), explored drug combinations, or had an expansion cohort were associated with even higher response rates.^2^, ^7^ More recently, owing to improvements in genomics and growing emphasis on precision‐based medicine, master protocols in the form of basket and umbrella trials have been increasingly employed to study targeted agents in cancer research. Basket trials are clinical studies investigating agent(s) targeting a common predictive risk factor (commonly a biomarker) across various tumor types, whereas umbrella trials test multiple targeted interventions in a single disease, which has been stratified into various subgroups based on different biomarkers or molecular signatures.^8^

The American Society of Clinical Oncology recently released a position statement on phase I trials, reiterating that, while remaining an integral part of clinical cancer research, these trials do indeed have therapeutic intent.^9^ Further reinforcing the importance of early phase trials, the US Food and Drug Administration in 2012 announced the “Breakthrough therapy designation for experimental drugs” to expedite the development of promising drugs based on preliminary clinical evidence.^10^, ^11^ Notable examples of drugs to benefit from this pathway are the programmed death receptor (PD‐1) targeting antibody pembrolizumab in melanoma, and the small molecule tyrosine kinase inhibitor ceritinib in non‐small lung cancer possessing the anaplastic lymphoma kinase gene rearrangement.^12^, ^13^ Both drugs went on to be granted accelerated approvals for their respective indications in 2014, less than 5 years after the first patient was enrolled in the corresponding phase I trial.^14^, ^15^, ^16^ While the expedited approval pathways do not apply to the majority of agents investigated in phase I trials, these examples illustrate that well‐designed phase I trials have the potential to streamline drug development and ultimately allow for earlier patient access to effective therapies.

Much of the published literature reporting on the trends and outcomes of phase I trials have taken place in the era of cytotoxic agents. Few reviews have included molecular targeted agents (MTAs) and immuno‐oncology (IO) agents, with even fewer addressing combination trials, thus failing to shed light on the most recent trends. Additionally, large systematic reviews of early phase trials rely on published results of trials and are therefore inherently prone to publication bias. The rate of unpublished trials is reported to be as high as 30%^17^ and this gap in the results could skew the overall interpretation of phase I trial outcomes. To better understand the evolving landscape of early phase drug development, we undertook a retrospective review of all phase I oncology trials enrolling patients over a 6‐year period between 2013 and 2018 at a single tertiary Australian center. We report on patient demographics, trial characteristics, safety, and treatment outcomes.

## 2 METHODS AND STATISTICAL ANALYSIS

Following approval from the Monash Health Human Research Ethics Committee, all adults with a solid organ malignancy screened for a phase I trial from January 1, 2013 to December 31, 2018 at Monash Health, Melbourne, Australia were identified from the medical oncology research database. Data collection from the hospital electronic medical records included baseline demographics, previous lines of treatment, type of investigational agent(s) (drug class), screening outcome, treatment response, and toxicity and survival outcomes. Investigational agents were classified as IO, MTAs, cytotoxic agents, antibody drug conjugates (ADCs), and other. For statistical analysis of clinical outcomes, trials were broadly grouped into two types—IO (if the study involved at least one immuno‐oncologic agent) and non‐IO. Trials were also categorized as single agent or combination treatments (trial category).

An early referral was defined as a patient referred in either the first‐ or second‐line setting for advanced disease. A late referral was defined as a patient referred after receiving more than two prior lines of systemic therapy. Screening visits refer to consultations at which patients signed the Patient Informed Consent Form for trial participation. Screen failure was defined as the inability of a consented patient to receive any study drug administration due to ineligibility, patient withdrawal of consent, decline in clinical status, or trial suspension (sponsor decision).

Treatment responses were collected from clinic notes and correlated with radiology reports. All trials utilized the Response Evaluation Criteria in Solid Tumors (RECIST) v1.1 criteria for response assessments. Prostate cancer trials additionally utilized the Prostate Cancer Working Group 2 response criteria. The response‐evaluable population was all patients who had at least one treatment response assessment. Toxicity grading was performed using the Common Terminology Criteria for Adverse Events (CTCAE) version 4 or 4.1 scale. Rates of clinically significant grade 2 (defined as toxicities that directly resulted in dose reduction, dose interruption, or study drug cessation) or grade 3–5 treatment‐related adverse events (TRAEs) were recorded. Toxicity data were determined for the population of patients that received at least one dose of study drug. Chi‐square (*χ* ^2^) testing was performed to detect any differences between ORR based on trial type and trial category. OS was defined as the time from consent to death from any cause. Kaplan–Meier estimates of survival were calculated separately for patients grouped by trial type and referral type (early vs late). Ninety‐day mortality (90DM) rates were calculated from the date of trial enrolment for the entire cohort. Statistical analysis was performed using IBM SPSS Statistics for Windows, Version 25.0. Armonk, New York.

## 3 RESULTS

**FIGURE 1:**
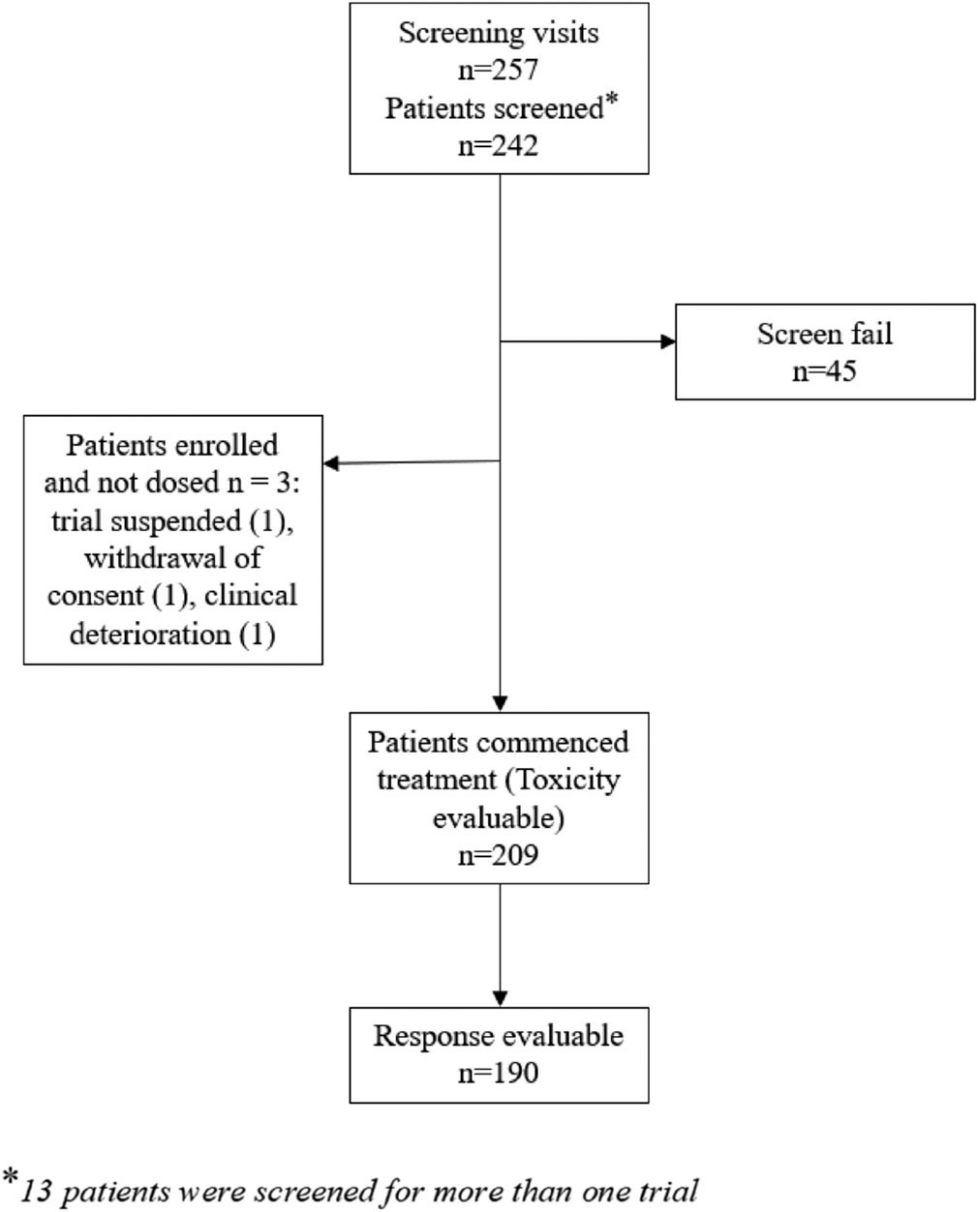
Screening and enrolment

Twenty‐eight phase I trials in solid tumors were conducted at our center over the 6‐year study period and 242 patients were screened (Figure 1). Thirteen patients were screened for more than one trial (including two patients who each screened for three different trials), yielding a total of 257 screening visits (Figure 1). Of these visits, there were 45 incidents of screen failure (18%). The most common reasons for screen failure were abnormal laboratory values out of the required range for eligibility (*n* = 14%, 31%) and deterioration in performance status prior to dosing despite fulfilling performance status criteria at screening (*n* = 8%, 18%). Other causes of ineligibility were secondary to protocol‐defined exclusions including comorbid illness (*n* = 3%, 7%), concurrent second malignancy (*n* = 3%, 7%), brain metastases (*n* = 2%, 4%), absence of measurable disease (*n* = 2%, 4%), absence of requisite biomarker(s) (*n* = 1%, 2%), prohibited concomitant medications (*n* = 1%, 2%), and prolonged corrected QT interval on baseline electrocardiogram (*n* = 1%, 2%). Three patients were enrolled but did not commence treatment. Therefore, the toxicity‐evaluable safety cohort of subjects who received a minimum of one dose of study drug consisted of 209 patients (86%). The response‐evaluable cohort consisted of 190 (79%) patients who had at least one response assessment.

Table 1 lists demographic details of screened patients. Median age was 64 years (range 30–89) and 50% (*n* = 121) of patients were male. The European Cooperative Oncology Group (ECOG) performance status of all patients was either 0 (*n* = 105, 44%) or 1 (137, 56%). Patients referred to the unit had a median of two lines of treatment in both time periods (range 0–12). The most frequently seen tumor types are also listed in Table 1.

**TABLE 1:** Patient demographics (*N* = 242)^a^

Characteristics	Total (%)
Median age (years)	64
Male	121 (50)
Female	121 (50)
Performance status	
0	105 (44)
1	137 (56)
Previous lines of systemic therapy	
0	37 (15)
1	84 (35)
2	50 (21)
3	34 (14)
4+	37 (15)
Referral source	
Internal	98 (41)
External	144 (59)
Tumor type	
Colorectal	29 (12)
Ovarian	28 (12)
Breast	25 (10)
SCLC	22 (9)
Mesothelioma	17 (7)
Bladder	16 (7)
Head and neck	14 (6)
NSCLC	11 (5)
Pancreas	11 (5)
Esophageal	11 (5)
Gastric	11 (5)
Cholangiocarcinoma	10 (4)
Prostate	10 (4)
Renal	9 (4)

Abbreviations: NSCLC, non‐small cell lung cancer; SCLC, small cell lung cancer.

^a^ 13 patients were subsequently screened for more than 1 trial; data pertaining to the first screening visit are presented.

### 3.1 Trial characteristics and recruitment

Of the 21 trials, eight (29%) were first‐in‐human (FIH). Most studies were histology‐agnostic while four were specific to tumor type (mesothelioma, small cell lung cancer, and two prostate cancer trials). Of the 28 trials, only 1 (4%) was investigator‐initiated, with the remaining 27 being industry‐sponsored. Four (14%) trials required the presence of a tissue‐based biomarker for study eligibility, which was confirmed by central laboratory assessment during a “pre‐screening” process; these included a BRAF V600E mutation (1), BRAF V600/KRAS/NRAS mutation (1), mesothelin positivity in the dose expansion phase (1) and HER2 positivity (1). Only one trial (4%), a study involving the combination of an oncolytic virus (administered via intrahepatic injection) and an immune checkpoint inhibitor mandated serial tumor biopsies at various time points. Fourteen trials investigated single agents only, nine were combination studies, and five studies had a monotherapy arm followed by combination treatment. The majority of IO agents investigated were immune checkpoint inhibitors; PD‐1/programmed death‐ligand 1 (PD‐L1) inhibitors as single agents or in combination were studied in 14 trials and one trial involved a bispecific antibody targeting PD‐1 and cytotoxic T‐lymphocyte‐associated protein 4 (CTLA‐4). Other drugs classified as IO included an adenosine receptor antagonist, an indoleamine‐pyrrole 2,3‐dioxygenase inhibitor, two oncolytic viruses and a bispecific antibody targeting CD3ε on T‐cells and prostate‐specific membrane antigen in refractory prostate cancer. MTAs included both small molecules and monoclonal antibodies with specific cancer‐relevant targets. Two trials featured ADCs, one targeting mesothelin in select solid tumors and the other targeting the HER2/ErbB2 receptor in tumors with HER2 overexpression and/or amplification. Two trials involved cytotoxic agents and one trial, classified as ‘other,’ investigated a novel iron chelator. The breakdown of recruitment based on trial type and category is described in Table 2.

**TABLE 2:** Patients enrolled to phase 1 trials (n = 209), according to drug class

Drug class	Total (%)
Single agent treatment	141 (67%)
MTA	59 (28%)
IO	45 (22%)
Cytotoxic	14 (7%)
ADC	19 (9%)
Other	4 (2%)
Combination treatment	68 (33%)
IO + MTA	32 (15%)
IO + IO	25 (12%)
IO + ADC	7 (3%)
Cytotoxic + MTA	3 (1%)
MTA + MTA	1 (1%)

Abbreviation: ADC, antibody drug conjugate; IO, immuno‐oncologic agent; MTA, molecular targeted agent.

### 3.2 Responses and survival

Of all patients (*n* = 209) who received at least one dose of trial‐specified treatment, 190 (91%) had a disease response assessment. Nineteen (9%) patients came off trial prior to the first scheduled response assessment scan due to the following reasons—early clinical progression (*n* = 10), cancer‐related death (*n* = 4), toxicity (*n* = 4) and unrelated medical illness (*n* = 1). Forty‐one (22%) patients had a confirmed response as defined by RECIST v1.1. Of these, 7 (4%) patients had a complete response, and 34 (18%) patients had a partial response. In addition, 59 (31%) subjects had stable disease producing a DCR of 53%. When grouped according to trial type, ORR in IO trials was 28% compared to 14% in non‐IO trials (*p* = .022). Patients in combination trials experienced a superior ORR than those treated in single‐agent studies (33% vs 16%; *p* = .005). Patients referred early had an ORR (24%) compared with those referred late (18%) (*p* = .257).

**FIGURE 2:**
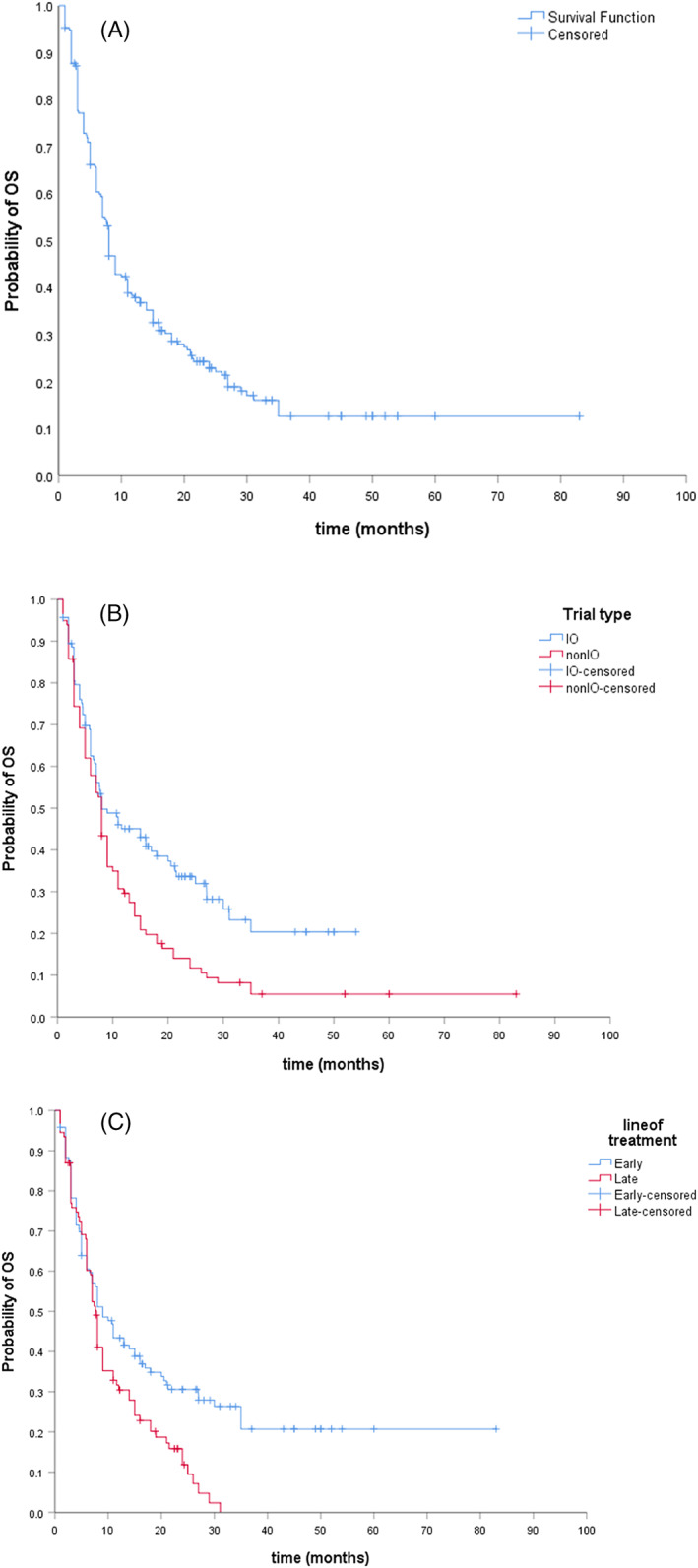
Kaplan–Meier curves for OS for the enrolled population (A), based on trial type (B) and line of therapy (C)

As of April 2020 (data cut‐off), 166 (78%) of all patients enrolled had died. After a median follow‐up of 23.2 months (range: 1.0–84.2), the median OS for the entire cohort was 8.0 months (95% CI: 6.8–9.1). Median OS calculated according to trial type was identical at 8 months in IO and non‐IO trials (*p* = .003). Furthermore, there was no difference in OS when comparing trial category (single agent vs combination; *p* = .132). The median OS in patients referred earlier was superior to those referred late (9.0 vs 7.8 months, *p* = .004) (Figure 2). The 90DM rate for the entire cohort was 20% (41 out of 209 enrolled subjects).

### 3.3 Toxicity

Clinically significant grade 2 and all ≥ grade 3 TRAEs and the corresponding trials by drug class are detailed in Table 3. Nineteen (9%) patients experienced clinically significant grade 2 TRAEs and 33 grade 3 or higher TRAEs were observed in 28 (13%) patients. Grade 4 toxicities accounted for less than 2% of all TRAEs. There was one treatment‐related death (0.5%), which occurred on a single agent ADC trial (pneumonitis). There were 13 (12%) incidences of clinically significant immune‐related adverse events (irAEs) across the 109 IO‐treated patients including 5 (5%) clinically significant grade 2 events and 8 (7%) grade 3 events. There were no reported grade 4 or 5 irAEs. There were no statistically significant differences in the incidence of grade 3 or higher TRAEs based on trial type (*p* = .07) or category (*p* = .83). Dose‐limiting toxicities (DLTs) at our center occurred in 3 (1%) patients, 2 of whom were on single agent MTA trials and 1 on a single agent ADC trial.

**TABLE 3:** Incidence of clinically significant TRAE's (*n* = 209)

Treatment‐related adverse event (TRAE)	Grade 2^a^	Grade 3	Grade 4/5	Trial category by drug class
	*n* = 19 (9%)	*n* = 28 (16%)	*n* = 5 (3%)	
Skin toxicity^b^	3			MTA
Fatigue	2	2		MTA
Gastro‐intestinal toxicity^c^	2	2	1	MTA
Myopathy		1		Cytotoxic
Febrile neutropenia		2	1	Cytotoxic, MTA
Neutropenia	2	2	1	Cytotoxic, MTA
Anemia	1			IO + MTA
Infusion reaction	1	4		Cytotoxic, MTA
Liver dysfunction (elevated transaminases)		1		ADC
Ascites		2		ADC, ADC + IO
Mucositis		1		ADC
Nephritis			1	MTA
Pneumonitis	1	1	1^d^	Cytotoxic, ADC
Neuropathy	2			Cytotoxic
Immune‐related adverse events				
Polymylagia rheumatica		1		IO + MTA
Gastritis	1			IO + IO
Immune‐mediated skin toxicity	2	1		IO + IO, IO + MTA
Hypophysitis	2			IO + MTA, IO
Colitis		2		IO
Hepatitis		4		IO + MTA
Myositis		1		IO + MTA
Encephalitis		1		IO

^a^ Clinically significantly Grade 2 treatment‐related adverse defined as events resulting in drug interruption, dose modification or study drug cessation.

^b^ Skin toxicity included Palmar‐Plantar Erythrodysethesiae (PPE) and 1 incidence of a photosensitive erythematous rash.

^c^ Gastro‐intestinal toxicity included nausea, vomiting, or diarrhea.

^d^ Grade 5 toxicity was a case of pneumonitis on an ADC (antibody‐drug conjugate) trial.

## 4 DISCUSSION

The principal objectives of phase I trials have conventionally been to characterize the safety profile of the agent(s) under investigation, and to establish the recommended phase II dose (RP2D) for further evaluation.^16^, ^18^, ^19^, ^20^ Traditionally, determination of the RP2D relies on careful evaluation of adverse events and utilizes a toxicity‐driven endpoint—the maximum tolerated dose (MTD).^1^, ^21^, ^22^ Importantly, this design was developed to investigate cytotoxic drugs, with the assumption that the dose‐toxicity and dose‐efficacy relationships are similar, resulting in a narrow therapeutic index.^21^, ^23^ However, novel anti‐cancer drugs with diverse mechanisms and toxicity profiles have challenged this paradigm.^1^, ^2^ A retrospective study of almost 700 patients enrolled on phase I trials of mostly MTAs at the MD Anderson Cancer Centre between 2004 and 2008 demonstrated similar efficacy outcomes across all dose levels (<25% MTD, 25%–75% MTD, and >75% MTD).^24^ Additionally, the cumulative toxicities of MTAs and late toxicities associated with IO drugs are poorly captured by the classical phase I trial designs.^1^

Efficacy endpoints such as ORR, progression‐free survival (PFS) and OS are often secondary endpoints due to relatively small numbers of patients recruited to early phase trials.^19^ Rates of response have historically been modest, which in turn has fueled the major criticism of phase I oncology trials—a debatable risk‐benefit ratio for patients enrolled.^18^, ^25^ Von Hoff et al reviewed 8000 patients over a period of 14 years from 1970 to 1983 and reported an overall response rate across all subjects of 6%.^26^ A subsequent review of all National Cancer Institute Cancer Evaluation Therapy Program conducted phase I trials between 1991 and 2002 revealed an ORR of 10.6%.^18^ With the rapid influx of newer treatments in oncology including MTAs, ADCs and most recently IO therapies, more recent reports describe response rates closer to 20%^7^ and assessment of efficacy in early phase trials has become increasingly pertinent. The IO agents, particularly anti‐PD‐1/PD‐L1 antibodies, have likely contributed to the improving response rates due to their ability to induce anti‐cancer immunity and durable anti‐tumor responses, albeit only in select patients and tumor types.^27^, ^28^, ^29^, ^30^, ^31^, ^32^

In our study, ORR was 22% and DCR was 53%, independent of drug class, comparable to that of recently published data.^7^ Also consistent with the trend in recent reviews,^7^, ^16^, ^33^ we observed only a small number of cytotoxic drug trials and a predominance of IO and combination trials, most notably in P2. Potential reasons proposed for the improving anti‐tumor activity seen in phase I trials have included the presence of expansion cohorts, biomarker‐driven trials, growing numbers of combination studies as well as more effective therapies.^2^, ^7^ One or more of these factors are applicable to most (80%) of the trials, we have conducted during this six‐year period and therefore could explain some of our findings. The median OS of our entire cohort was 8.0 months, comparable to previously reported survival on phase I trials of 8–10 months.^19^, ^34^, ^35^ It is interesting to note that although the median OS between IO and non‐IO trials was the same, there was a late separation of the curves, which may be driven by the durability of responses that are commonly associated with IO therapies.

The growing success of phase I trials has encouraged referral for earlier participation as a therapeutic option as opposed to a last resort; the early referral rate at our institution may reflect this trend, where almost half of all patients (47%) were referred either untreated for advanced disease or after only one line of systemic therapy. We can speculate that trials investigating IO and MTAs were attractive to referrers, and such studies were already starting to feature by 2013, when our study period commenced. Additionally, phase I trials in our unit provided an opportunity for patients to access anti‐PD‐1/PD‐L1 drugs in the absence of drug approval and government reimbursement, likely contributing to earlier referral patterns. We found that patients referred early also had an improved OS compared with those referred later. Although OS is typically longer in earlier lines of therapy for approved agents or combinations in many tumor types, the longer OS seen in the phase I setting from our cohort is potentially a reflection that agents from drug classes with proven activity were being employed.

A screen failure rate of 18% compared favorably to the previously reported rate of 25% in phase I trials.^36^ The leading causes of screen failure at our center were similar to those in the published literature, namely, out‐of‐range laboratory values and the deterioration of health prior to dosing. Although screen failures are inevitable, the relatively low rates we observed may have been in part due to the proportion of early referrals when patients are typically more robust and retain a better performance status, as well as appropriate patient selection prior to the screening process.

The issue of risk and potential harm associated with phase I trials in oncology has long been debated.^18^, ^22^, ^25^ Our study revealed relatively low rates of high grade TRAEs and only one treatment‐related death. These findings demonstrate the relative safety of phase I trial enrolment. The incidence of irAEs in the IO trials was low with no Grade 4 or 5 events in the setting of stringent guidelines for the early detection and management of irAEs. It is important however to note that early recognition and effective management of TRAE's, particularly irAE's, would have improved over the 6‐year period with the institute's growing trial portfolio—this could partly account for the favorable safety profile observed.

This study has clear limitations including its retrospective nature and single center focus. Our relatively small cohort makes it difficult to draw conclusions relating to safety and anti‐tumor activity. Additionally, the trials in our portfolio were heterogenous involving a variety of trial designs and investigational agents with distinct mechanisms of action. There were only four basket trials that is, investigating a biomarker‐directed therapy across different tumor histologies, and no umbrella trials—perhaps reflecting the time period of the study. These master protocols have lately emerged as critical tools in investigating targeted therapies and data pertaining to their influence on early phase clinical research would ideally feature in a study of this kind. Nevertheless, the major strength of our study is its real‐world representation of individual patient data. There is certainly a recognized need to share and access patient‐level phase I trial data in order to optimize trial design, identify important safety issues and ultimately improve patient care.^37^ Previous systematic reviews of trends in phase I oncology trials have been criticized due to inherent publication bias as they drew results from PubMed searches. Consequently, the response rates reported could possibly be an overestimate of the true result. A future registry‐based database would be of great value to monitor trends and outcomes in the dynamic field of early drug development.

In conclusion, our study adds to the growing body of evidence supporting phase I oncology trials as valid treatment options. It highlights the complexities surrounding design, endpoints, biomarker use, and clinical outcome reporting. Notably, there is a paucity of such data in an Australian context and hence the findings of this study are unique and valuable when considering the evolving phase I trial landscape in oncology. The 90DM rate of 20% in a good performance status group highlights the poor prognosis for most patients with advanced solid organ cancer and hence it is incumbent on clinicians to exercise caution while conducting early phase trials by carefully consenting patients and offering reasonable expectations based on pre‐clinical and clinical evidence. Finally, as next generation sequencing and other forms of biomarker identification become more prevalent, the role of optimal patient selection when conducting early phase oncology trials will become increasingly relevant.

## CONFLICT OF INTEREST

Dr Ben Markman—Novartis advisory board member, Amgen advisory board member. Prof Eva Segelov—MSD advisory board member, IPSEN advisory board member, speaker fees from SHIRE, fees from MERCK SERONO. The other authors do not have any conflicts of interest or funding sources to disclose.

## AUTHOR CONTRIBUTIONS

All authors had full access to the data in the study and take responsibility for the integrity of the data and the accuracy of the data analysis. *Conceptualization*: B.M, D.D, S.M.; *Data curation*: S.M., A.D.; *Methodology*: B.M, D.D, S.M.; *Investigation*: S.M, A.D, B.M, D.D.; *Resources*: S.M, A.D, C.H.; *Writing—Original Draft*: S.M, A.D, D.D, B.M.; *Writing—Review and Editing*: all authors; *Visualization*: S.M, B.M, D.D.; *Supervision*: B.M, D.D.; *Funding acquisition*, *project administration*, *software*, *validation*: Not applicable.

## ETHICS STATEMENT

The study was conducted following approval from the Monash Health Human Research Ethics Committee (Ref: RES‐19‐0000‐953Q). Requirement for patient consent: Not applicable.

## Data Availability

The data that support the findings of this study are available on request from the corresponding author. The data are not publicly available due to privacy or ethical restrictions.
